# A novel approach to improve immune effector responses post transplant by restoration of CCL21 expression

**DOI:** 10.1371/journal.pone.0193461

**Published:** 2018-04-04

**Authors:** Heather E. Stefanski, Leslie Jonart, Emily Goren, James J. Mulé, Bruce R. Blazar

**Affiliations:** 1 Department of Pediatrics, University of Minnesota, Minneapolis, Minnesota, United States of America; 2 Cutaneous Oncology Program, Moffitt Cancer Center, Tampa, Florida, United States of America; Maisonneuve-Rosemont Hospital, CANADA

## Abstract

Chemotherapy or chemoradiotherapy conditioning regimens required for bone marrow transplantation (BMT) cause significant morbidity and mortality as a result of insufficient immune surveillance mechanisms leading to increased risks of infection and tumor recurrence. Such conditioning causes host stromal cell injury, impairing restoration of the central (thymus) and peripheral (spleen and lymph node) T cell compartments and slow immune reconstitution. The chemokine, CCL21, produced by host stromal cells, recruits T- and B-cells that provide lymphotoxin mediated instructive signals to stromal cells for lymphoid organogenesis. Moreover, T- and B-cell recruitment into these sites is required for optimal adaptive immune responses to pathogens and tumor antigens. Previously, we reported that CCL21 was markedly reduced in secondary lymphoid organs of transplanted animals. Here, we utilized adenoviral CCL21 gene transduced dendritic cells (DC/CCL21) given by footpad injections as a novel approach to restore CCL21 expression in secondary lymphoid organs post-transplant. CCL21 expression in secondary lymphoid organs reached levels of naïve controls and resulted in increased T cell trafficking to draining lymph nodes (LNs). An increase in both lymphoid tissue inducer cells and the B cell chemokine CXCL13 known to be important in LN formation was observed. Strikingly, only mice vaccinated with DC/CCL21 loaded with bacterial, viral or tumor antigens and not recipients of DC/control adenovirus loaded cells or no DCs had a marked increase in the systemic clearance of pathogens (bacteria; virus) and leukemia cells. Because DC/CCL21 vaccines have been tested in clinical trials for patients with lung cancer and melanoma, our studies provide the foundation for future trials of DC/CCL21 vaccination in patients receiving pre-transplant conditioning regimens.

## Introduction

Bone marrow transplant (BMT) is a life-saving modality used to treat malignant and nonmalignant disorders. Chemoradiotherapy conditioning, that precedes donor graft infusion, damages thymic and LN stroma, severely delaying peripheral CD4^+^ and CD8^+^ T cell reconstitution [1–3]. The endogenous T cell response is defective for 6–24 months post-transplant [2, 4–8]. Thus, BMT recipients are at increased risk of opportunistic fungal and viral infections [4, 6, 7, 9, 10]. Moreover, recent clinical evidence has shown higher relative CD4 and CD8 counts in patients with chronic lymphocytic leukemia (CLL) are independent predictors for survival, emphasizing the importance of immune reconstitution in survival [11]. Strategies to increase these responses early post-transplant by augmenting thymopoiesis or peripheral T cell expansion in BMT patients have been unable to fully restore a functional immune system [12–14].

We and others published that although exogenous addition of Keratinocyte Growth Factor (KGF) results in supranormal thymopoiesis in mice post-BMT by stimulating thymic epithelial cell proliferation, mature thymic-derived T cells recently migrating from the thymus into the periphery remained profoundly depleted [15–18]. These studies led to the hypothesis that the prolonged duration of T cell lymphopenia seen in patients after myeloablated BMT is not solely reflective of thymus involution and injury, which has been the existing paradigm in the field. In support of this hypothesis, antigen-specific T cell infusion to treat solid or hematopoietic malignancies can have variable efficacy even in the context of partial or full myeloablative conditioning, which induces pro-inflammatory cytokines, antigen release, lymphopenia, and homeostatic expansion of infused and endogenous T cells [19, 20]. While initial expansion occurs, we hypothesize that endogenous and perhaps adoptively transferred T cell therapies may be limited by radiation-induced lymph node (LN) injury which causes mislocalization of T cells into non-lymphoid organs. The effector T cells that find their way into non-lymphoid organs may then fail to receive survival signals resulting in suboptimal immune responses.

In BMT recipients, the LN is small and disorganized; host fibroblastic reticular cells, critical for antigen transport in the LN and spleen, are depleted [3, 21–23]. In addition there is a paucity of expression of key chemokines within secondary lymphoid organs needed for T- and B-cell recruitment into these sites, including CXCL13 and CCL21. CXCL13, produced by T cells and LN stroma, is selectively chemotactic for CXCR5+ B cells (both B-1 and B-2 subsets)[24, 25]. CXCL13 controls the organization of B cells within lymphoid follicles and is expressed highly in the LNs, spleen, GI tract and liver on high endothelial venules, along with CCL19 and CCL21 [26, 27]. The essential role of CXCL13 has been reported in the establishment and maintenance of lymphoid tissue microarchitecture.

CCL21 is one of the mediators of CCR7 signaling and is found throughout the paracortical sector of the LN; CCL21 is secreted by stromal cells, high endothelial venule cells and lymphatic endothelial cells as well [28, 29]. CCR7 signaling is critical for migration of mature antigen presenting cells (APC) to the LN and naïve T cell extravasation from blood to LNs through the high endothelial venules [30, 31]. We first reported that CCL21 expression was markedly reduced in secondary lymphoid organs of BMT recipients [3]. We also found that fibroblast reticular cell (FRC) numbers were depleted after BMT [3]; both CCL21 and FRCs provide key homeostatic signals to naïve T cells [32, 33]. We further showed that a p53 inhibitor given 30 minutes prior to radiation limited stromal cell injury, partially restoring CCL21 protein levels and improving LN architecture. These data have led us to hypothesize that selectively providing CCL21 protein could improve immune effector responses to both pathogens and tumor in a lethal radiation congenic model of BMT.

## Materials and methods

### Animals

C57BL/6 (H-2b; termed B6) female mice were purchased from The Jackson Laboratory (Bar Harbor, ME) and used at 8 weeks of age as BMT recipients or control animals (non-BMT control mice). Donor female C57BL/6.Ly5.1 mice of the same age were purchased from the National Cancer Institute (Frederick, MD). Mice were housed in specific pathogen-free facilities. The IACUC and IBC Committees at the University of Minnesota approved all protocols.

Mice with evidence of being moribund (which is defined as non-responsive to gentle stimulation) were killed and scored as dead. Mice that lost more than 20% of their original weight (after recovery from irradiation) were considered clinically moribund (University of Minnesota) and were also killed and scored as dead. Alternatives to these types of experiments have been explored, but after discussion with experts in the field it was determined that these survival in vivo experiments were the best to answer these critical questions post transplant. Mice are assessed at least daily, and weighed twice weekly. Once the mouse was determined to be moribund, it was sacrificed at that time. The duration of the tumor experiment is 100 days; mice were sacrificed at that time point if they had not succumbed to disease. 50% of animals died of disease prior to being sacrificed. The mice that died or were sacrificed died of disease. The number of animals used per group was 4–6 for at least two experiments. To reduce stress in animals, gentle mouse handling techniques are stressed. Lab members handling mice must be proficient and fast at injections. Confinement in x-ray jigs is minimized. Eye ointment is used during anesthesia to prevent corneal drying (during bioluminescent imaging). The new shredded paper nesting material is more suitable to sick mice. Fecal balls are removed from the rectums of mice with diarrhea with warm moist gauze pads to prevent irritation of the skin. All personnel are trained by Research Animal Resources (RAR) and need to undergo additional training by laboratory-trained personnel. There are SOPs for all mice procedures in the lab that must be followed.

### Bone marrow transplantation

Single-cell suspensions of BM cells obtained from femurs and tibiae of B6.Ly5.1 (congenic) donors were CD4/8-depleted as described previously [34] and 5 × 10^6^ (congenic) CD4/8-depleted BM cells were intravenously administered to recipients that had received 11-Gy TBI from a cesium source 24 h before BMT.

### Generation of dendritic cells (DCs)

Erythrocyte-depleted bone marrow cells flushed from the femurs and tibias of B6 mice were cultured in 10 ng/ml GM-CSF and 10 ng/ml IL-4 (R and D Systems, Minneapolis, MN) at 1 × 10^6^ cells/ml in CM (RPMI 1640 containing 10% heat-inactivated FCS, 0.1 mM nonessential amino acids, 1 μm sodium pyruvate, 2 mM fresh L-glutamine, 100 μg/ml streptomycin, 100 units/ml penicillin, 50 μg/ml gentamicin, 0.5 μg/ml fungizone, and 5 × 10^−5^ M 2-mercaptoethanol). At day 3, fresh cytokines were added, and nonadherent cells were harvested on days 5–7 by gentle pipetting. DCs were enriched by density centrifugation over 14.5% (w/v) matrizamide (Sigma Chemical Co., St. Louis, MO). The low-density population was washed once in CM and once in RPMI 1640 containing 2% FCS prior to use. The resulting DC population was >85% positive for coexpression of MHC II, CD11c, CD40, CD80, and CD86 (data not shown).

### Genetic modification of DCs with adenoviral vectors and injection into mice

DCs were resuspended at a concentration of 1 × 10^7^ cells/ml in RPMI 1640 + 2% FCS and placed in a 15-ml conical tube. Adenoviral-Null was purchased from Vector Biolabs, Malvern, PA and Adenoviral-CCL21 was purchased from Vector Biolabs, Malvern, PA or received from the NIH Repository, Bethesda, MD. The virus was added at a ratio of 20,000 vector particles/DC, the suspension was mixed well, and the tube was incubated at 37°C for 2 h. Nine volumes of complete medium with 10 ng/ml GM-CSF and 10 ng/ml IL-4 were then added, and the cells were transferred to tissue culture dishes. For pathogenic responses, the DCs were also pulsed with lysates of tumor, bacteria or virus depending on the experiment. Cells were incubated for 18 h at 37°C, supernatants were recovered, and the cells were purified by incubation in PBS with 3 mM EDTA and gentle scraping. Supernatants were collected and frozen; the CCL21 ELISA (R and D systems) was performed and CCL21 was quantified in pg/ml. The cells were washed several times in PBS, resuspended to 1 × 10^7^ cells/ml. 1 x 10^6^ cells were injected intra-muscularly the left hind paw on days 21, 28 and 35 post transplants.

### Lymphocyte flow cytometry

Splenocytes, and LNs were suspended in 2% fetal calf serum/phosphate-buffered saline (PBS), and 10^6^ cells were incubated with appropriate fluorochrome-conjugated monoclonal antibodies (BD Pharmingen, San Jose, CA) for 30 minutes at 4°C. A total of 10^5^ live events were acquired on a Fortessa flow cytometer (BD Pharmingen) and analyzed with FlowJo software (TreeStar, San Jose, CA).

### Confocal microscopy

Intact spleens and LNs were embedded in optimum cutting temperature (OCT) compound (Sakura, Tokyo, Japan) and were snap-frozen in liquid nitrogen and stored at −80°C. For LN/spleen analysis, 6-μm cryosections were acetone-fixed and stained for CCL21 (R&D Systems) or CXCL13 (R and D systems) along with B220-FITC (clone RA3-6B2; eBioscience) and CD8a Cy5 (clone 53–6.7, eBioscience) for 3 hours at room temperature. CCL21 signals were amplified with Tyramide Signal Amplification kit according to the manufacturer’s instructions (Invitrogen). Slides were mounted with VECTASHIELD (Vector Laboratories) and images were acquired through a 10×/0.40 Olympus UPlanApo or 40×/0.80 Olympus UPlanApo Oil lens and an Olympus FV500 camera, compiled with Fluoview software (v.4.3), then analyzed and cropped in Adobe Photoshop CS2.

### *Listeria monocytogenes* infection

The recombinant *L monocytogenes* strain ΔactA-*Lm*-OVA (attenuated) expressing full-length chicken ovalbumin was kindly provided by Dr. S. S. Way (University of Minnesota). Mice were inoculated with early logarithmic phase (OD600 of 0.1) bacteria grown in brain heart infusion broth at 37°C. Mice were injected intravenously with 10^6^ colony-forming units (CFU) of ΔactA-*Lm*-OVA diluted in 200 μL PBS [10].

### Quantification of Lm-OVA-specific CD8 T cells

MHC-I-DimerX:mouse-Ig-PE was purchased from BD Biosciences, and purified OVA257-64 (SIINFEKL) peptide was purchased from Anaspec (San Jose, CA). MHC-I-DimerX:mouse-Ig:OVA257-64 conjugates were prepared according to manufacturer’s instructions (BD Biosciences). Single cell suspensions of lymph node and spleen were incubated with DimerX:mouse-Ig:OVA257-64-PE plus antibodies for other markers (all from BD Biosciences), and 5 × 10^3^ donor CD8 T cells were collected and analyzed by flow cytometry.

### Determination of *L monocytogenes* CFU

Eight days after infection, livers were removed and homogenized in 0.05% Triton X-100/PBS (Sigma-Aldrich). Serial dilutions were plated onto brain-heart infusion plates, and *Lm* colonies were enumerated after 24 to 48 hours at 37°C.

### Determination of vesicular stomatitis virus plaque forming units

1 × 10^5^ pfu vesicular stomatitis virus (VSV) strain Indiana (i.v. injection) were given on day 42. Mice were examined on day 43 post infection [35]. Spleens were harvested and a single-cell suspension was prepared after RBC lysis. The plaque assay was performed as previously described [36].

### Acute myelogenous leukemia cell line and survival

C1498FFDsR, stable transfectants of C1498 (an AML cell line obtained from ATCC) that express the fluorescent *Discoma* coral-derived protein DsRed2 and firefly luciferase, were prepared [37]. C1498FFDsR (10^6^) was injected into transplanted animals on day 42 post transplant into the tail vein. Survival was monitored weekly.

### Statistical analysis

The Kaplan-Meier method of survival analysis was used to display overall survival, and the log-rank test was used to evaluate the difference in survival distributions between comparison groups. One-way ANOVA with post-hoc Tukey test and Student *t* test unpaired comparison were used to determine significant differences between each group presented as bar graphs using the Graph Pad Prism software. Results are presented as means ± standard error; *P* values < .05 were considered to be significant.

## Results

### DC/CCL21 vaccination restores chemokine expression post-BMT

We recently found a paucity of total and naïve donor T cells in the LN despite normal or supranormal thymopoiesis in transplant recipients that was associated with a lack of T (CCL21) and B (CXCL13) cell attracting chemokine expression in the secondary lymphoid organs [3]. This led to the hypothesis that CCL21 deficiency in host stromal cells would preclude the recruitment of recent thymic emigrants to secondary lymphoid organs.

To test our hypothesis, we adopted a vaccine strategy to exogenously increase CCL21 expression in the secondary lymphoid organs. Previously, we showed that use of an adenoviral CCL21 gene modified DC-based tumor vaccine could enhance T cell recruitment in vivo [38, 39]. We adapted DC/CCL21 based vaccines to our BMT model. DCs generated from mouse BM were infected with either adenoviral CCL21 (DC/CCL21) or an adenovirus devoid of CCL21 (termed null virus; DC/null). DC/CCL21 and DC/null produced 1,920–4,008 and 57–253 pg/ml/10^6^ cells/24 hours, respectively (p<0.05, data not shown). C57BL/6 mice were lethally irradiated, rescued with congenic T cell depleted BM, and given DC/CCL21 or DC/null vaccines intramuscularly beginning 21 days post-transplant and then weekly for three total doses. Mice were sacrificed at day 50 for analysis.

**Fig 1:**
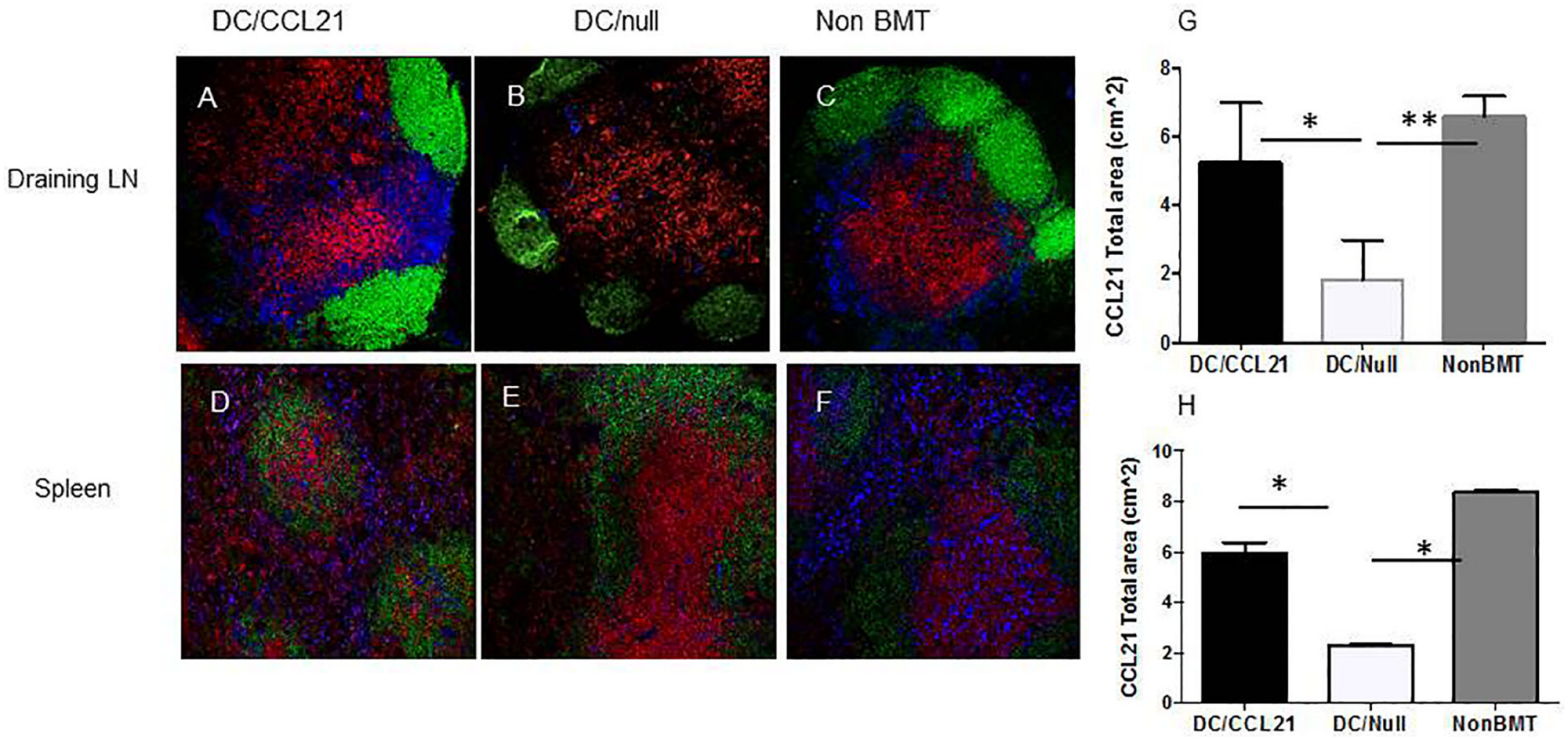
CCL21 expression is improved in mice post-DC/CCL21 vaccination. Immunofluorescence staining of LNs and spleen was performed on day 50 after transplant to assess for CCL21 (blue), B cells (B220) (green) and T cells (CD8a)(red). A-C and G are draining LN; D-F and H are spleen. G-H are plots of CCL21 area in the draining lymph node and spleen respectively using Image J. This is a representative of at least 3 animals per group; this experiment was performed two times. * = p<0.05 ** = <0.01.

We first sought to determine if CCL21 expression was increased in mice that had received DC/CCL21. In mice that received the DC/null vaccine, there was similar expression to BMT only mice (data not shown). As shown in Fig 1, DC/null mice had decreased expression of CCL21 compared to the non-BMT control in the draining LNs and the spleen (Fig 1B versus Fig 1C and Fig 1E versus Fig 1F respectively). Importantly, when DC/CCL21 vaccine was given, CCL21 expression was restored to normal levels compared to the non-BMT mice (Fig 1A vs. 1C and 1D vs. 1F). CCL21 expression was also substantially more intense compared to mice that received the DC/null vaccine (Fig 1A vs. 1B and 1D vs. 1E). Quantitation using Image J to determine the CCL21 total area revealed significantly higher CCL21 expression in non-BMT animals compared to DC/null (P<0.01) and in DC/CCL21 animals compared to DC/null (p<0.05) in the draining LN (Fig 1G). There was no difference between the non-BMT mice control group and the DC/CCL21 group. In the spleen (Fig 1H), CCL21 expression was much greater in non-BMT animals compared to DC/null (P<0.05) and in DC/CCL21 animals compared to DC/null (p<0.05). This shows that although the injection was localized to the hind paw, CCL21 expression was increased in the draining LN (Fig 1A and 1G) as well as both the spleen (Fig 1D and 1H) and non-draining LN (data not shown). Moreover, expression of CCL21 was focused in the T cell areas (Fig 1A and 1D) in mice that had received DC/CCL21. CCL21 expression in the T cell areas is critical for naïve and memory cell entry through the high endothelial venules into the lymph nodes in order to mount an immune response [40].

### DC/CCL21 vaccination results in restoration of both CXCL13 expression and LTi cells

**Fig 2:**
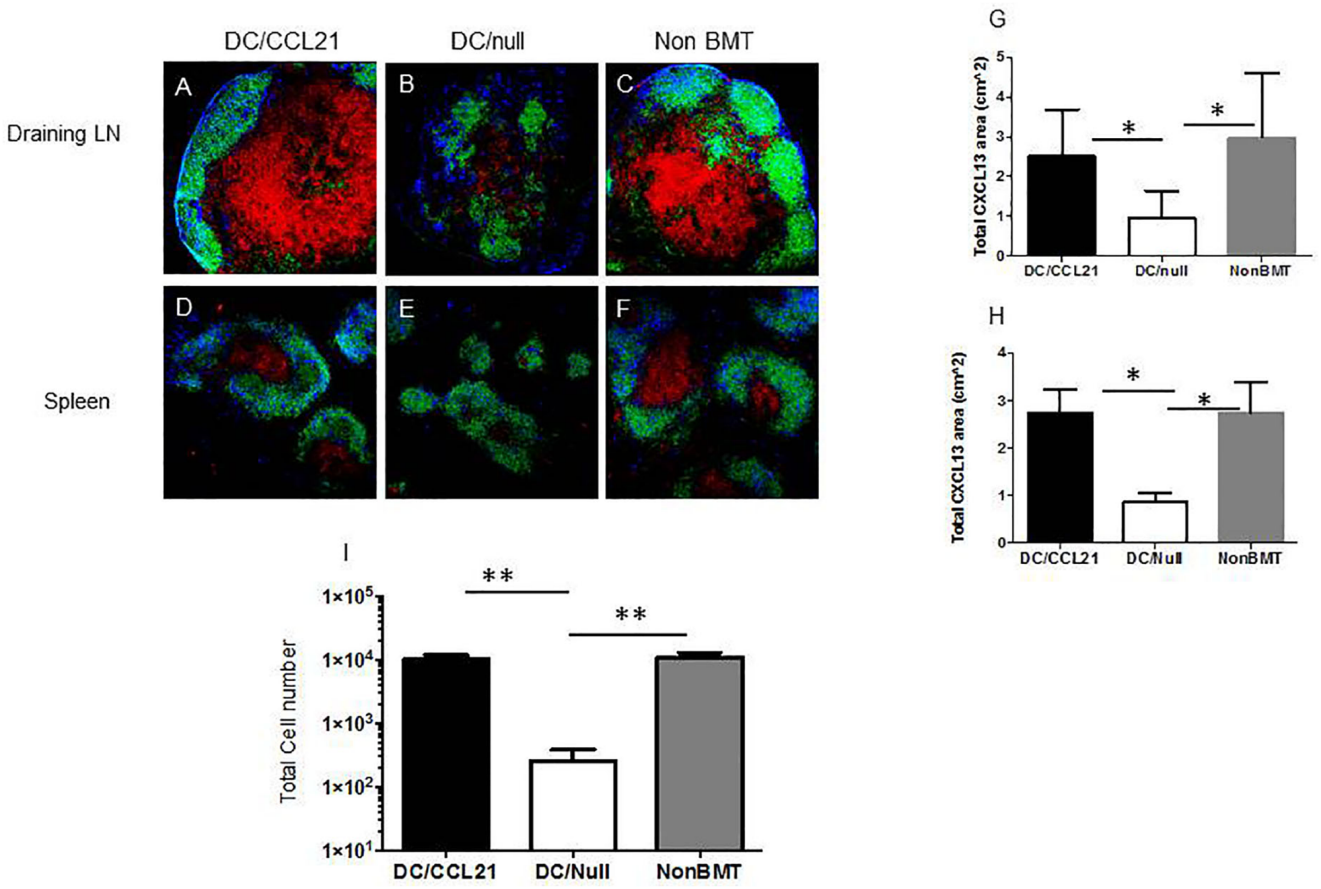
CXCL13 expression and LTi cells are improved in mice that received DC/CCL21. Immunofluorescence staining of LNs and spleen was performed on day 50 after transplant to assess for CXCL13 (blue), B cells (B220) (shown in green) and T cells (CD8a)(shown in red). A-C shows draining LN; D-F shows spleen. Representative area of CXCL13 is shown in G, H for draining LN and spleen, respectively. The number of LTi cells (donor-derived, CD4^+^CD3−CD11c−B220−) cells in the draining LN is shown in I. There were 3–5 animals per group; this is representative of two experiments. * = p<0.05 ** = <0.01.

Chemotherapy and radiation also causes decreased CXCL13 expression and B cell areas in the secondary lymphoid organs [3]. Because DC/CCL21 injection had improved T cell chemokine expression, we sought to investigate whether a similar principle held true for B cell areas in the spleen and LN. As shown in Fig 2, DC/null mice had decreased expression of CXCL13 compared to the non-BMT control in the draining LNs and spleen (Fig 2B versus Fig 2C and Fig 2E versus Fig 2F). Interestingly, when DC/CCL21 vaccine was given, CXCL13 expression was restored to normal levels in draining LNs and spleen compared to the non-BMT mice (Fig 2A vs. 2C and 2D vs. 2F), as confirmed by Image J analysis (Fig 2G and 2H). CXCL13 expression was also significantly (p<0.05) greater compared to BMT mice that received DC/CCL21 vs. the DC/null vaccine (Fig 2A vs. 2B and 2D vs. 2E; Fig 2G and 2H).

There was no significant difference between the non-BMT mice control group and the DC/CCL21 group in either organ. Moreover, the expression of CXCL13 was within the B cell area of both the LN and the spleen suggesting that DC/CCL21 vaccine is responsible for the reorganization of the LN.

Accumulation of lymphoid tissue-inducer (LTi) cells in secondary lymphoid organs correlates with preferential restoration of the lymphoid stromal compartment after injury (e.g., viral infection or chronic infection)[41]. In order for LNs to form, there is cross-talk between both LTi cells and mesenchymal organizer cells [42]. Early in development, LTi cells start to cluster at sites of nascent LN anlagen and are crucial for LN formation. RORγt^−/−^ mice do not have any LTi cells, which prevents both organizer cell and subsequent LN development in these mice [43]. LTi cells express lymphotoxin (LT) α1β2 and engage the LTβR present on organizer cells. This LTαβ/LTβR interaction is required for LN development and is demonstrated by the fact that LTα^−/−^, LTβ^−/−^, and LTβR^−/−^ mice lack all peripheral LNs [44]. The signaling between the LTi and organizer cells through the LTβR induces expression of adhesion molecules and chemokines resulting in the recruitment of more LTi cells to the nascent LN anlage. Our previous work showed a paucity of LTi cells post-transplant that can be partially restored by KGF and p53 inhibitor administration [3]. Therefore, we next determined if DC/CCL21 vaccination could increase the number of LTi cells in our BMT model. As shown in Fig 2I, mice that received DC/Null vaccination, had ~30-fold fewer LTi cells in the draining LN compared to the non-BMT control (p<0.01), while mice that received DC/CCL21 had comparable LTi numbers as non-BMT controls. Taken together, our data shows that DC/CCL21 vaccination culminates in LN restoration of critical chemokines required to recruit lymphocytes to the lymph node.

### DC/CCL21 vaccination results in CD8^+^ T cell recruitment to the draining LN post BMT

**Fig 3:**
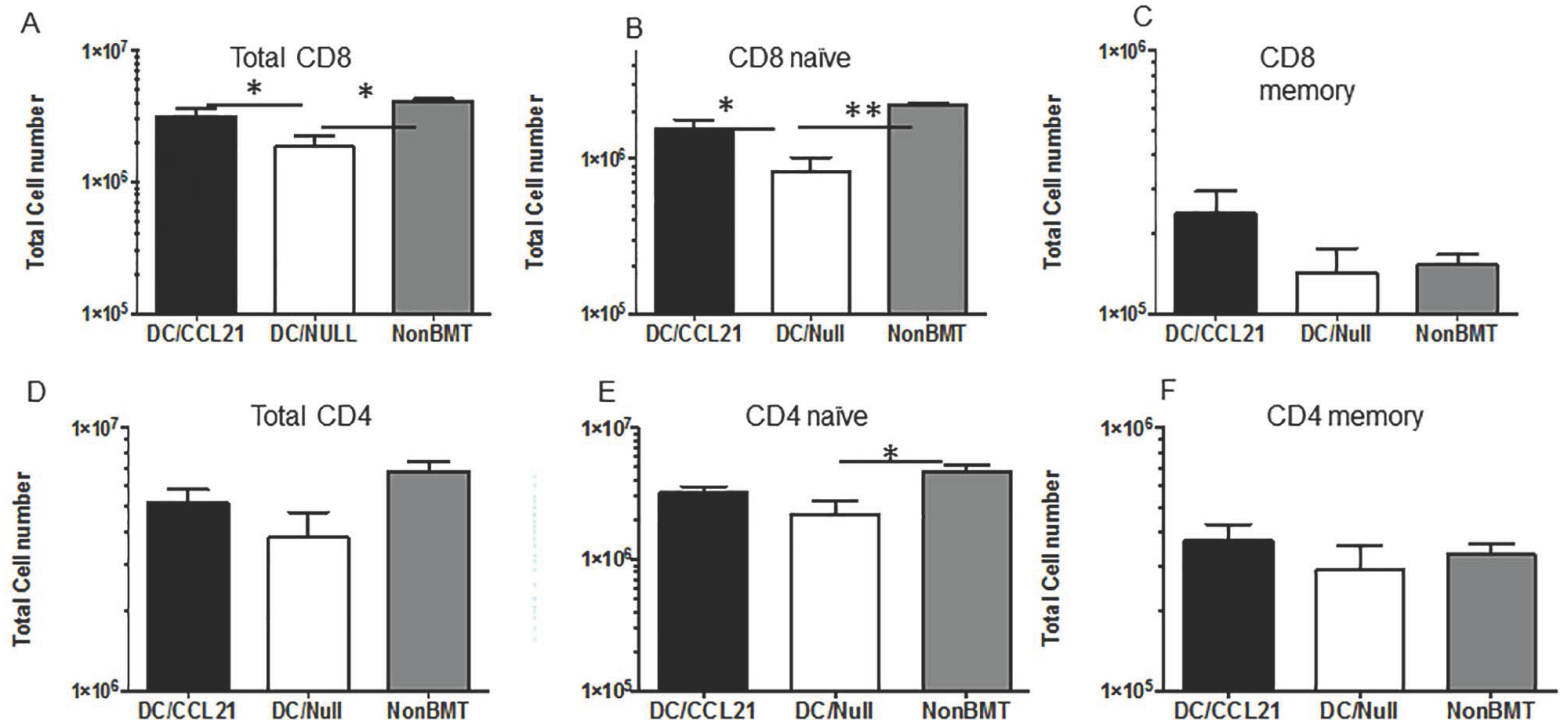
DC/CCL21 increases naïve CD8^+^ T cells in the draining LN On day 50 after transplant, draining lymph nodes (A-F) were stained for the presence of T cells. Mean absolute numbers ± SEMs of (A) donor-derived CD8^+^T cells; (B) donor-derived, naive (CD62LhighCD44low) CD8^+^ T cells; (C) donor-derived, memory (CD62LlowCD44hi) CD8^+^ T cells; (D) donor-derived CD4^+^T cells; (E) donor-derived, naive (CD62LhighCD44low) CD4^+^ T cells; (F) donor-derived, memory (CD62LlowCD44hi) CD4^+^ T cells; There were 3–5 animals per group; this is representative of two experiments. * = p<0.05 ** = <0.01.

Our previous data showed a paucity of CD8^+^ T cells in the secondary lymphoid organs post-transplant associated with decreased CCL21 expression [3]. We next determined if CD8^+^ T cells were recruited to the secondary lymphoid organs that had received the DC/CCL21 vaccine. As shown in Fig 3, the total CD8 cell number was significantly decreased by over 2-fold in mice that received DC/null compared to the non-BMT controls in draining LNs. However, there was a substantial increase in CD8^+^ T cell number in mice that had received DC/CCL21 compared to DC/null by 2-fold. In fact the CD8 cell number in the DC/CCL21 vaccinated mice was similar to the non-BMT controls (Fig 3A). Subset analysis was performed for naïve and memory CD8+ T cells. DC/null vaccinated mice had 2 fold fewer CD8 naïve cells compared to the non-BMT control. In contrast, DC/CCL21 vaccinated mice had 2 fold higher CD8 naïve cell numbers compared to the DC/null mice resulting in similar numbers of CD8 naïve between DC/CCL21 and non-BMT controls (Fig 3B). In the CD8 memory compartment, there were no significant differences based on vaccination (Fig 3C). As shown in Fig 3D and 3F, there are no differences in total CD4 or CD4 memory cells based on vaccination. However, in the CD4 naïve compartment, there is a 2-fold reduction of CD4 naïve cells in mice that received DC/Null vaccination compared to non-BMT controls with no difference between mice vaccinated with DC/CCL21 compared to non-BMT control mice.

**Fig 4:**
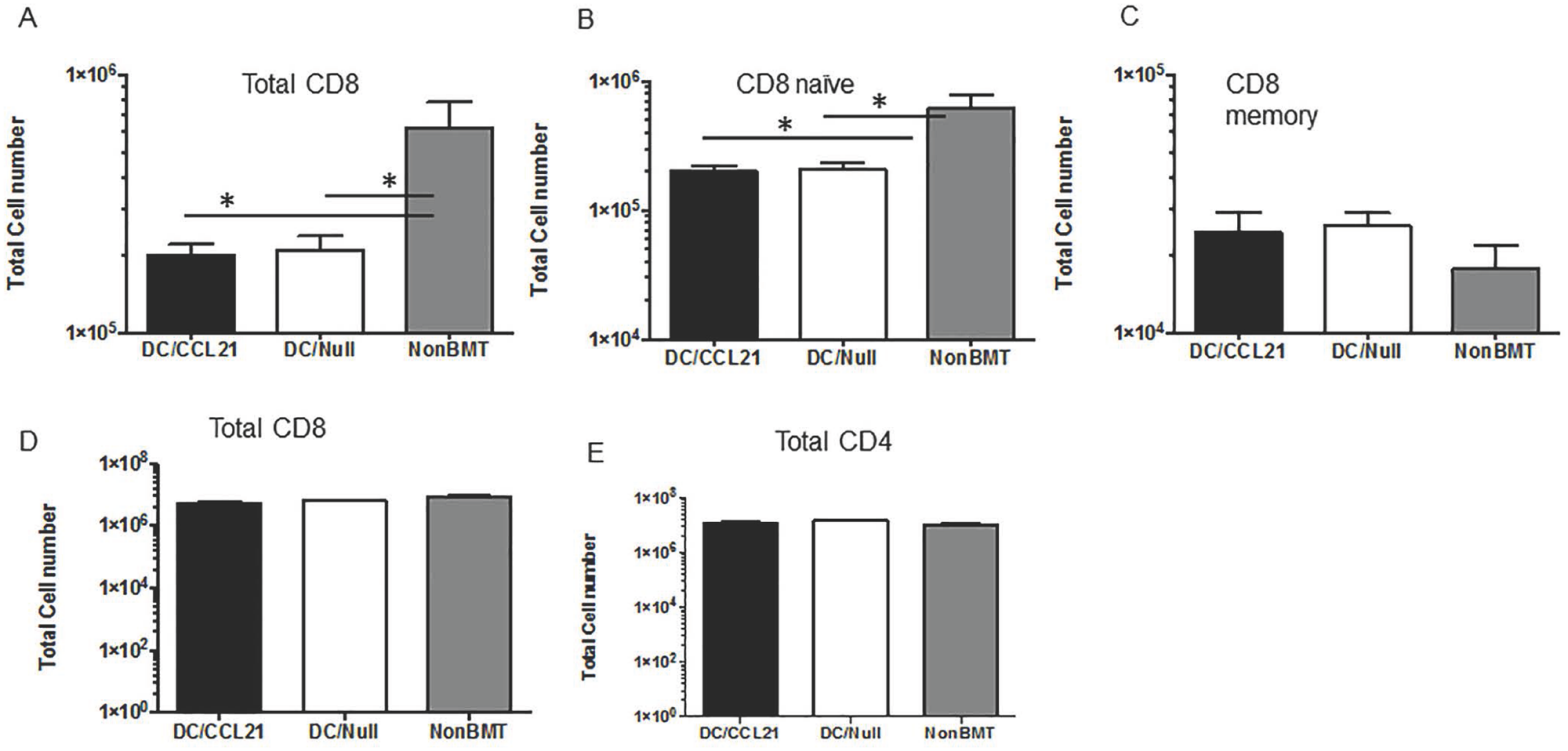
Despite restoration of CCL21 expression in peripheral lymphoid organs, T cell numbers were not increased. On day 50 after transplant, non- draining LNs (A-C) and spleens (D, E) were stained for the presence of T cells. Mean absolute numbers ± SEMs of (A) donor-derived CD8^+^T cells; (B) donor-derived, naive (CD62LhighCD44low) CD8^+^ T cells; (C) donor-derived, memory (CD62LlowCD44hi) CD8^+^ T cells; (D) donor-derived CD8^+^T cells; (E) donor-derived CD4^+^ T cells; There were 3–5 animals per group; this is representative of two experiments. * = p<0.05.

Although CCL21 expression was restored in DC/CCL21 injected mice to similar levels as the non-BMT control in non-draining LNs, increased donor-derived CD8 and CD8 naïve cells were not observed in this compartment. As shown in Fig 4A, DC/Null vaccination resulted in a 5-fold decrease in CD8 cells compared to non-BMT controls. In mice that received DC/CCL21, there was also a 5-fold decrease in CD8 cells compared to non-BMT controls. In fact mice vaccinated with CCL21 or null had no differences in total or naïve CD8+ T cell number (Fig 4A and 4B, respectively). Similar to the draining LN, there were no differences based on vaccination in the memory compartment (Fig 4C). In the spleen, there were no differences in any of the groups in regards to total CD8 or total CD4 cell number (Fig 4D and 4E, respectively). These data suggest that there are other important effects of the localized vaccine that may result in increased T cell homing to the draining LN.

### Pathogenic responses are improved in mice receiving DC/CCL21 vaccine

Taken together, our data have shown that DC/CCL21 improves expression of both T (CCL21) and B (CXCL13) cell selective chemokines. Our data also have shown increased T recruitment to the draining LN along with restoration of secondary lymphoid architecture. Therefore, we next sought to determine whether improved T cell reconstitution induced by DC/CCL21 vaccination would permit a functional immune response to challenge with a live intracellular pathogen.

**Fig 5:**
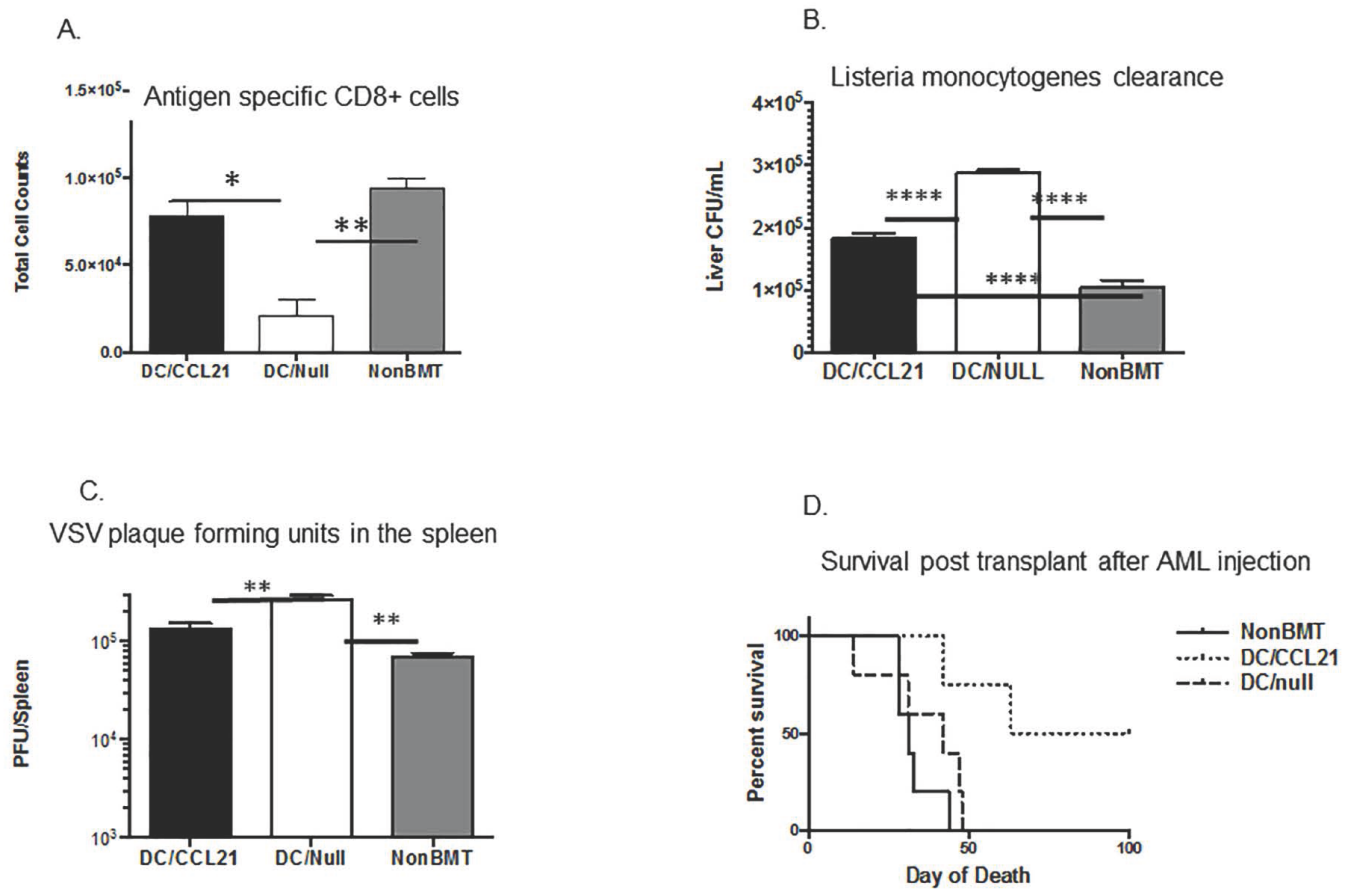
Pathogenic responses are improved in transplanted mice that received DC/CCL21. **A. Ova specific responses. BMT m**ice were assessed on day 50 for absolute numbers OVA–specific T cells. **B. Bacterial clearance**. To determine bacterial clearance, livers from BMT mice were assessed for bacterial clearance on day 50. Each group had 3–5 mice; this experiment was done with three replicates. **C. Viral clearance**. Spleens from BMT mice were assessed for viral clearance on day 43 [45]. Each group had 3–5 mice; this experiment was reproduced two times with similar results. **D. Anti-tumor responses**. BMT mice were injected on day 42 with tumor cells; survival was monitored weekly. There were at least 4 mice per group. This is representative of two experiments. P = 0.0009 utilizing log rank Mantel-Cox test.

We first tested mice against the bacteria *Listeria monocytogenes (Lm)*. Mice were immunized with 10^6^ CFU of an attenuated strain of *Lm* engineered to express the nominal antigen, chicken ovalbumin (Δ*actA-Lm-OVA*) on day 42 post-transplant, one week after the last DC vaccination. Eight days later, liver bacterial clearance was assessed and CD8 antigen specific responses were determined. In mice that received DC/null challenge, there was a 4-fold reduction in numbers of antigen specific CD8 cells compared to the non-BMT control (Fig 5A, p<0.01). Interestingly there was a 3.5-fold increase in the numbers of ova specific CD8 cells in mice that received DC/CCL21 compared to DC/Null mice (p<0.05) with no difference in ova specific responses between DC/CCL21 vaccination and non-BMT controls (Fig 5A). This antigen specific response correlated to the bacterial clearance in mice. In mice that received DC/null vaccine, there was a 3-fold increase in bacterial colonies compared to non-BMT controls and a 2-fold increase compared to DC/CCL21 vaccination along with a significant, 2-fold difference in bacterial clearance between mice that received DC/CCL21 and non-BMT controls (Fig 5B).

Next, we tested mice for responses to viral challenge. We utilized VSV in our model and determined clearance of virus 24 hours post infection. In mice that received DC/null vaccine, there was a 3- fold increase in plaque forming units in the spleen compared to non-BMT controls (P<0.01). There was also a 2-fold increase in plaque forming units in DC/null mice compared to DC/CCL21 vaccination (p<0.01) and no significant difference in viral clearance between mice that received DC/CCL21 and non-BMT controls (Fig 5C).

### Anti- tumor responses are improved in mice receiving DC/CCL21 vaccine pulsed with tumor lysates

In addition to deficiencies in pathogen clearance, BMT recipients may succumb to their underlying malignancy, ascribed in part to poor immune surveillance post-transplant [46]. To determine whether improved immune function could enhance the endogenous CD8^+^ T cell response to AML challenge post-BMT, congeneic BMT recipients were vaccinated with DC/CCL21 or DC/null pre-loaded with AML (C1498 cell) lysates. All mice received a lethal dose (10^6^) of C57BL/6 AML cells expressing firefly luciferase on day 42 post-BMT. Compared to the uniform lethality in both DC/null vaccine and non-vaccinated, non-BMT controls, mice receiving DC/CCL21 had a significant increase in survival with 50% of mice surviving long-term (Fig 5D, P = 0.0009). These data provide critical proof-of-concept that secondary lymphoid stromal injury repair conferred by AML lysate pulsed DC/CCL21 vaccines can harness the endogenous T cell immune response to eliminate progressive AML without requiring donor lymphocyte infusions or other post-BMT therapies. Moreover, this shows the importance of a DC-based vaccine in eliciting a functional tumor effector response.

## Discussion

In this report, we have employed a novel approach utilizing a vaccine model to restore CCL21 expression in BMT recipients. Local DC/CCL21 vaccination not only restored CCL21 expression in the draining lymph node, but also in the distant lymph nodes and spleen. Moreover, DC/CCL21 vaccination resulted in increased LTi cells and CXCL13 expression, restoring lymph node architecture. This lead to the ability of CD8 naïve cells to home to the draining LN. The most profound finding is that pathogenic responses and anti-tumor responses were observed post BMT, which is critical for survival. These data emphasize the importance of functional secondary lymphoid organs post BMT in order to achieve immunity.

The expression of chemoattractant receptors on leukocytes direct their migration to specific areas within the secondary lymphoid organs that are critical for appropriate antigen presentation [19, 31]. In particular, CCL21 chemokine gradients play an important role in recruiting T cells to secondary lymphoid tissues and direct the navigation and trafficking of T cells within secondary lymphoid organs in CCL21 deficient mice. In the draining LNs of DC/CCL21 challenged mice, we found an increase in CCL21, increase in CD8 naïve T cells, increase in LTi numbers and CXCL13 expression. We hypothesize that the draining LN in this model had either a physiological or supra-physiological CCL21 gradient resulting in more efficient T cell migration. Sources of CCL21 can include FRCs, high endothelial venules and other endothelial cells. In preliminary studies, we have found a 3-fold increase in FRC network in mice that received DC/CCL21 (data not shown). It is well known that FRCs are a rich cytokine source and can expand several fold in size in response to antigen [47]. It has also been shown that FRC expansion is dependent on trapping of naïve lymphocytes in the draining LNs [47]. Taken together, one could envision that DC/CCL21 vaccination initially restores CCL21 expression. This then recruits naïve T cells to the secondary lymphoid organs, which can induce FRC expansion thereby recruiting additional cell types and restoring CXCL13 expression in the B cell area.

Additional experiments by Zhang and colleagues recently showed that homing of progenitors to the thymus was significantly decreased in irradiated animals [48]. They found that there is a reduction in the chemokine CCL25 in mice that undergo radiation. In order to circumvent this defect, they pre-treated BM cells with CCL21 and found that this rescued profound T-lineage progenitor homing to the thymus in BMT recipients. Taken together, these data suggest that chemokine restoration post-radiation is critical for T cell homing to both the thymus and secondary lymphoid organs.

Recent work from Marchesi and colleagues have shown that overexpression of CXCL13 in the gut promoted the accumulation of IL-22 producing LTi cells [49], supporting our data indicating that CXCL13 expression is associated with increased LTi cells. Others have found that ectopic expression of CXCL13 in pancreatic islets results in the accumulation of both B and T cells and the formation of tertiary lymphoid structures [50–53]. In recent work by Dudakov and Hanash, IL-22 producing LTi cells in the thymus and gut were found to be relatively radiation resistant [52, 53]. The most likely explanation for our findings is that increased LTi cells are recruited to the LN and produced lymphotoxin signals critical for LN regeneration, thereby permitting CXCL13 expression to be restored.

Based on our data, one could envision the following: DC/CCL21 vaccine restores CCL21 expression resulting in recruitment of naïve T cells to the LN; the trapped naïve T cells can then increase the FRC network; the naïve T cell also provide lymphotoxin beta receptor signals which are critical in lymph node formation [54]; this results in the FRC network secreting additional cytokines and chemokines and recruiting other types of cells (B cells, LTi cells, more T cells) to the lymph node. Thus the LN is able to mount a functional immune response to pathogens and tumor.

Currently infections and relapse post-BMT are the lynch pins of successful treatment of hematopoietic tumors, such as AML. It has been shown in a number of studies that a functional immune system post-BMT is directly correlated to lower relapse rates [13]. Studies have evaluated whether T cells were capable of responding to different Herpes viruses (CMV, HHV6, HSV, EBV) in patients who underwent BMT [55, 56]. For example, patients who had responsive T cells had significantly fewer relapses, emphasizing the importance of a functional immune response [46]. Our data show that restoration of chemokines and improved cell trafficking to secondary lymphoid organs post-BMT result in improved immune function, thereby optimizing donor T cell recovery post-BMT. Moreover, this approach also may improve the efficacy of adoptively transferred T cells used to treat relapse post-BMT in the increasingly aged BMT recipient and in patients with known LN injury (e.g. HIV patients with LN fibrosis) whose immune system may benefit from LN regeneration approaches [57]. In a recent Phase I study, an autologous vaccination strategy was utilized to induce the generation of leukemic-specific T cells in patients undergoing reduced intensity transplant for CLL [58]. In this case, bystander cells secreting GM-CSF were utilized as the adjuvant. This study showed only a modest impact on recovering T cell populations with most of the CD8^+^ T cells being antigen-specific to CLL. Our current model has the potential to have a broader impact on immune reconstitution, as the antigen-specific T cells represented only a portion of the CD8+ T cells. Moreover, restoration of CCL21 resulted in CXCL13 restoration and increased LTi cells as well. One could envision CCL21 being part of post transplant therapy when immune reconstitution is critical for viral clearance and anti-tumor immunity; thus CCL21 could bolster the immune response resulting in decreased morbidity and mortality. Whether or not the findings reported herein hold promise for potentially changing the practice of BMT will need to await their translation into the clinic.
